# Recombinant HLA-G as Tolerogenic Immunomodulant in Experimental Small Bowel Transplantation

**DOI:** 10.1371/journal.pone.0158907

**Published:** 2016-07-12

**Authors:** Martin W. von Websky, Koji Kitamura, Isis Ludwig-Portugall, Christian Kurts, Maximilian von Laffert, Joel LeMaoult, Edgardo D. Carosella, Kareem Abu-Elmagd, Joerg C. Kalff, Nico Schäfer

**Affiliations:** 1 Department of Surgery, University Hospital of Bonn, Bonn, Germany; 2 Department of Hepatobiliary Pancreatic Surgery and Transplantation, Kyoto University Hospital, Kyoto, Japan; 3 Institute for Experimental Immunology, University of Bonn, Germany; 4 Charité Berlin, Institute of Pathology, Berlin, Germany; 5 CEA, iMETI, Research Division in Hematology and Immunology, Saint*-*Louis Hospital, Paris, France; 6 Transplant Center, Cleveland Clinic, Cleveland, Ohio, United States of America; INSERM-Université Paris-Sud, FRANCE

## Abstract

The non-classical MHC I paralogue HLA-G is expressed by cytotrophoblast cells and implicated with fetomaternal tolerance by downregulating the maternal adaptive and innate immune response against the fetus. HLA-G expression correlates with favorable graft outcome in humans and recently promising immunosuppressive effects of therapeutic HLA-G in experimental transplantation (skin allograft acceptance) were shown. Consequently, we examined this novel therapeutic approach in solid organ transplantation. In this study, therapeutic recombinant HLA-G5 was evaluated for the first time in a solid organ model of acute rejection (ACR) after orthotopic intestinal transplantation (ITX). Allogenic ITX was performed in rats (Brown Norway to Lewis) with and without HLA-G treatment. It was found that HLA-G treatment significantly reduced histologically proven ACR at both an early and late postoperative timepoint (POD 4/7), concomitant to a functionally preserved graft contractility at POD 7. Interestingly, graft infiltration by myeloperoxidase+ cells was significantly reduced at POD7 by HLA-G treatment. Moreover, HLA-G treatment showed an effect on the allogenic T-cell immune response as assessed by flow cytometry: The influx of recipient-derived CD8^+^ T-cells into the graft mesenteric lymphnodes at POD7 was significantly reduced while CD4^+^ populations were not affected. As a potential mechanism of action, an induction of T-reg populations in the mesenteric lymphnodes was postulated, but flow cytometric analysis of classical CD4^+^/CD25^+^/FoxP3^+^T_reg_-cells showed no significant alteration by HLA-G treatment. The novel therapeutic approach using recombinant HLA-G5 reported herein demonstrates a significant immunosuppressive effect in this model of allogenic experimental intestinal transplantation. This effect may be mediated via inhibition of recipient-derived CD8^+^ T-cell populations either directly or by induction of non-classical T_reg_ populations.

## Introduction

In intestinal transplantation, episodes of ACR are frequent and impact severely on patient survival and outcome. While ACR may be treated by intensifying baseline immunosuppression and administration of pulsed steroids, concomitant systemic inflammatory response and sepsis due to compromised intestinal barrier function under increased immunosuppression increases mortality. In the past, attempts to achieve functional (or “prope”) tolerance in intestinal transplantation to minimize potentially harmful over-immunosupression and improve long-term outcome by avoiding chronic rejection have not been successful. Historic strategies, like the enhancement of bone-marrow chimerism or the depletion of passenger lymphocytes via graft irradiation, were abandoned after initial enthusiasm [1,2]. Meanwhile, induction therapy with depleting antibodies, calcineurin-inhibitors and steroids have become the workhorse in intestinal transplantation with excellent short and midterm outcome in specialized centers [2]. Nevertheless, this approach has failed to achieve “prope tolerance”, even though some patients can be successfully weaned to very low doses of immunosupression without developing rejection [2,3]. Chronic graft dysfunction (observed in up to 20% of patients) remains the major obstacle of intestinal transplantation and new tolerogenic strategies are needed where conventional immunosuppression has failed. Therefore, a new strategy to counteract rejection after intestinal transplantation was tested in this study. The non-classical MHC I paralogue HLA-G has been implicated with a central role in fetomaternal tolerance and could be a promising potential candidate for tolerogenic protocols in solid organ transplantation [4]. Expressed mainly–but not exclusively- by the human cytotrophoblast, it partakes in the protection of the fetus (e.g. a semi-allogenic allograft) from the mother’s immune system via downregulating adaptive (T cell) and innate (NK cell) immunity [5,6]. The observation that HLA-G was expressed on immunoprivileged tissues [7,8] and could effectively downregulate T- and NK-cell immune responses as well as DC maturation in vitro [9,10], has stimulated transplant-related research: Higher HLA-G expression levels in heart, liver, kidney and lung transplant recipients correlated with favorable outcome and fewer rejection episodes [11,12]. While this approach hinted at involvement of endogenous HLA-G expression in transplant-patient subgroups with favorable outcome, it did not test the efficacy of HLA-G as a potential therapeutic agent to counteract allograft rejection. Recently, promising studies have shown tolerogenic effects of exogenic HLA-G when used as a therapeutic immunomodulant in dimeric form allowing application in experimental transplantation. Favier et al. have demonstrated skin allograft acceptance in mice after intraperitoneal administration of HLA-G coated polystyrene beads [13]. Because of the known mechanisms of action of HLA-G (e.g. blunting the CD8^+^ T-cell response or inducing tolerogenic regulatory T-cells [14]), it was hypothesized that soluble HLA-G would exhibit similar tolerogenic properties in experimental intestinal transplantation. Thus, in this study, HLA-G was evaluated in experimental intestinal transplantation—a solid organ especially prone to acute and chronic rejection.

## Material and Methods

### Study design

**Fig 1:**
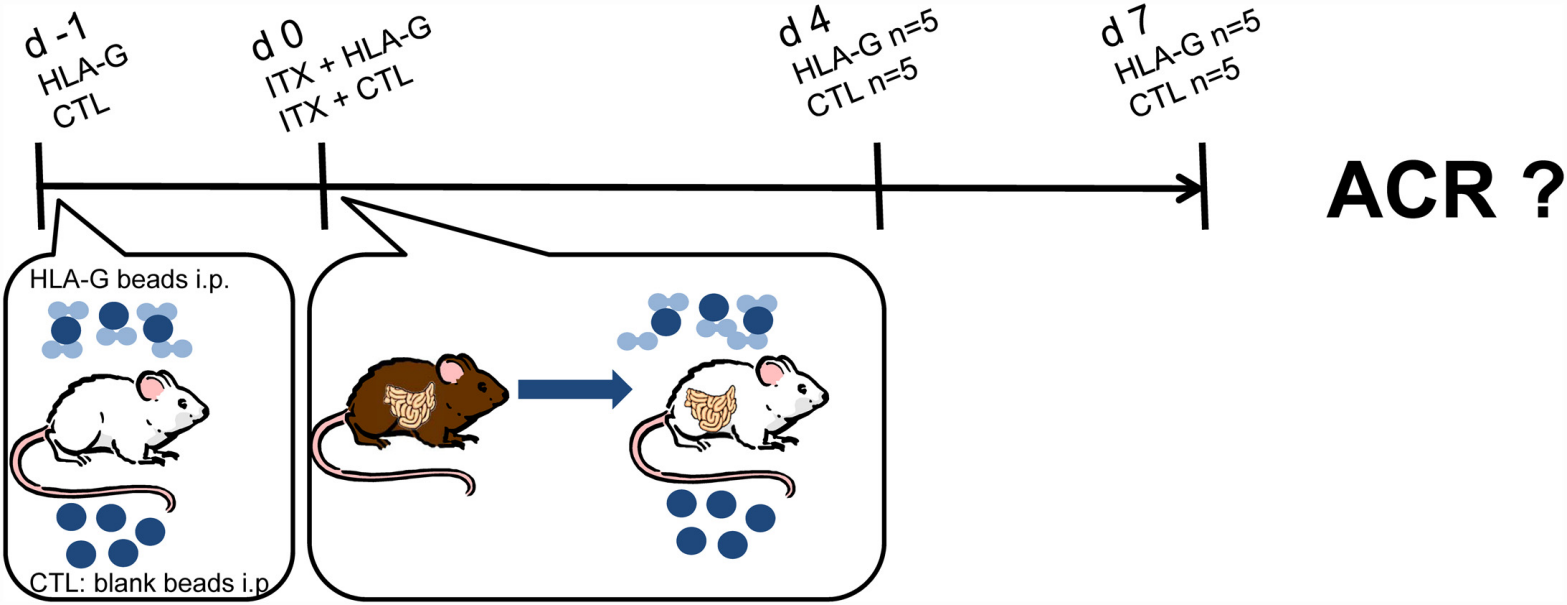
Experimental groups and study design.

To study the therapeutic potential of recombinant HLA-G to prevent rejection of intestinal grafts, orthotopic allogenic ITX (Brown Norway (BN) to Lewis (LEW)) was performed in 4 groups (n = 5 rats each) using a standardized technique with end-to-side aorto-aortic and porto-caval microvascular anastomoses and reestablishment of enteral continuity. A cold and warm ischemia time of 60 and 30 minutes respectively was maintained during the operative procedure and University of Wisconsin solution was used for organ preservation during cold ischemia, as previously described [15]. The high responder strain combination (BN->LEW) is known to exhibit severe ACR after 4–7 days without immunosuppression after intestinal transplantation, and accordingly these time points were chosen for evaluation of ACR as optimal to detect the postulated immunosuppressive effect [16]. For the study, the two groups of recipient animals (LEW) received 10 million of HLA-G coated polystyrene beads i.p. at 24h hours prior to and upon completion of the transplant procedure (see Fig 1). The control groups received the same amount of blank polystyrene beads without prior HLA-G loading. The animals were then observed for four or seven days before sacrifice and assessment of ACR. Survival experiments where not planned or performed due to german animal protection regulations. For a synopsis of the experimental design and groups see Fig 1.

### Animals

Inbred male LEW and BN rats weighing 180–200 g were obtained from Charles River WIGA GmbH (Sulzfeld, Germany). All experiments were performed in accordance with the european and german federal law regarding the protection of animals with prior approval by the Landesumweltamt and principles of laboratory animal care (NIH Publication No.8 5–23, revised 1985) were followed. All surgery was performed under isoflurane inhalation anesthesia with additional carprofen analgesia (5mg /kg s.c for 3 days), and all efforts were made to minimize suffering. Animals were kept at the facility for seven days prior to the procedure for acclimatization and maintained on a 12-h light/dark cycle and provided with commercially available chow (Altromin, Lage, Germany) and tap water ad libitum, respectively. At the end of the respective observation periods animals were euthanized under general anesthesia with isoflurane overdose and exsanguination during organ procurement.

### Immunoprecipitation of HLA-G / HLA-G beads

Cell supernatant containing HLA-G, or more specifically, the ß2-microglobulin fused to the HLA-G5 heavy chain (referred to as B2M-HLA-G5), was generously supplied from Dr. Le Maoult's group and coated on 4.5μm polystyrene beads (Polysciences Inc., Eppelheim, Germany) as previously described [13]: In short, the polystyrene beads were first incubated with anti-HLA-G antibody 5A6 (Exbio, Praha) overnight in 0.1 M borate buffer. In a second step, the antibody-coated beads where used for immunoprecipitation of B2M-HLA-G5 from the previously mentioned cell supernatant. A western blot using the diagnostic anti-HLA-G antibody clone 4H84 (Exbio, Praha) confirmed loading of the beads with the fusion protein. Control beads were prepared in the same manner using PBS instead of cell supernatant containing B2M-HLA-G5.

### Flow cytometric analysis

Graft draining lymphnodes were sampled and analyzed by flow cytometry using a BDCANTOII flow cytometer (BD Franklin Lakes, New Jersey USA) after lymphocyte isolation. Antibodies used for FACS-staining for T-cell analysis were biotinylated anti-rat CD45 (Biolegend, San Diego, USA) with secondary streptavidin-Pacific Blue (Life Technologies, Darmstadt, Germany), anti-rat CD4 PerCP-eFluor 710 (eBioscience, San Diego, USA) and anti-rat CD8 APC (eBioscience, San Diego, USA). To distinguish between recipient (LEW) and donor (BN) derived T-lymphocytes (i.e “passenger lymphocytes”), a monoclonal anti-rat MHC class I antibody (specific for BN MHC class I haplotype “c”, RT1.Ac, AbD Serotec), was used. Antibodies used for analysis of T_reg_ were anti-rat CD45 and anti-rat CD4 as above, followed by anti-rat CD25 APC (eBioscience, San Diego USA) and anti-FoxP3 FITC (eBioscience, San Diego USA) using the FoxP3 staining buffer set (eBioscience, San Diego USA) according to manufacturer’s description. Samples were evaluated using a standardized gating strategy with FlowJo Version 10 (Treestar Inc., Ashland USA). For analysis, relative T cell proportions are given as percentages of the respective CD45^+^ parent population. This strategy, with CD45^+^ primary staining as a pan-leucocytic marker was chosen to ensure comparability of the results, despite possible variation in efficiency of leucocyte isolation.

### Real time quantitative PCR

For the gene expression analysis, several known ACR-related markers were chosen. Our group has shown previously that TNFα, IFNγ, ICAM-1, IL-10, IL-6 overexpression in the tunica muscularis is associated with ACR in this model of orthotopic allogenic intestinal transplantation [17]. For the expression analyses, mRNA was isolated from sampled mid-jejunal graft muscularis (RNeasy kit, Qiagen, Hilden,Germany), transcribed to cDNA (HighCapacity cDNA reverse transcription, Life Technologies, Darmstadt, Germany) using kits according to manufacturer’s instructions and measured on a Taqman cycler (7900 HT, Life Technologies, Darmstadt, Germany) for real-time PCR. For the evaluated cytokines (TNFα, IFNγ, ICAM-1, IL-10, IL-6), FAM-labeled probes were obtained (Life Technologies, Darmstadt) and the housekeeping gene GapDH was used for normalization. To establish the presence of the PIR-B receptor (Lilrb3/NILR-1, Entrez Gene ID 65146) in the graft mesenteric lymphnodes (gMLN) in our model, RT-PCR was likewise performed with a specific FAM-labeled probe against the respective mRNA with a 77bp amplicon (Thermofisher Scientific Assay No: RN00581823). The presence of the specific amplicon was confirmed by gel staining (see S2 Fig).

### Statistics

Data was analyzed with GraphPad Prism 5.04 (La Jolla, USA). After testing for normal distribution using the D’Agostino and Pearson’s tests, the appropriate methods of analysis were determined. For non-normally distributed data, non-parametric testing (Mann-Whitney U / Kruskal-Wallis test) followed by Dunn’s multiple comparison’s test was employed. For normally distributed data, student’s t-test or one-way ANOVA was performed. P values < 0.05 were considered significant. Interrater reliability of histopathologic assessment was confirmed using Cohen’s κ. As described by Landis and Koch 1977, Cohen’s κ was interpreted as “slight agreement” for the range of 0–0.20, “fair agreement” for the range of 0.21–0.40, “moderate agreement” for the range of 0.41–0.60 and “substantial agreement” for the range of κ = 0.61–0.80 [18]. Smooth muscle contractility, as measured in a dose-response curve using the muscarinic agonist betanechol in a standard organ bath, was compared by fitting the normalized data to a logistic curve (four-parameter sigmoidal model) and comparing the fitted midpoint (logEC_50_) and slope, using the F test and Graph Pad Prism 5.04. All data relevant to reach the conclusions drawn in the manuscript with related metadata and methods, and any additional data required to replicate the reported study findings in their entirety is found in the manuscript and supporting information files.

## Results

### Assessment of acute rejection by histology (Wu-score)

**Fig 2:**
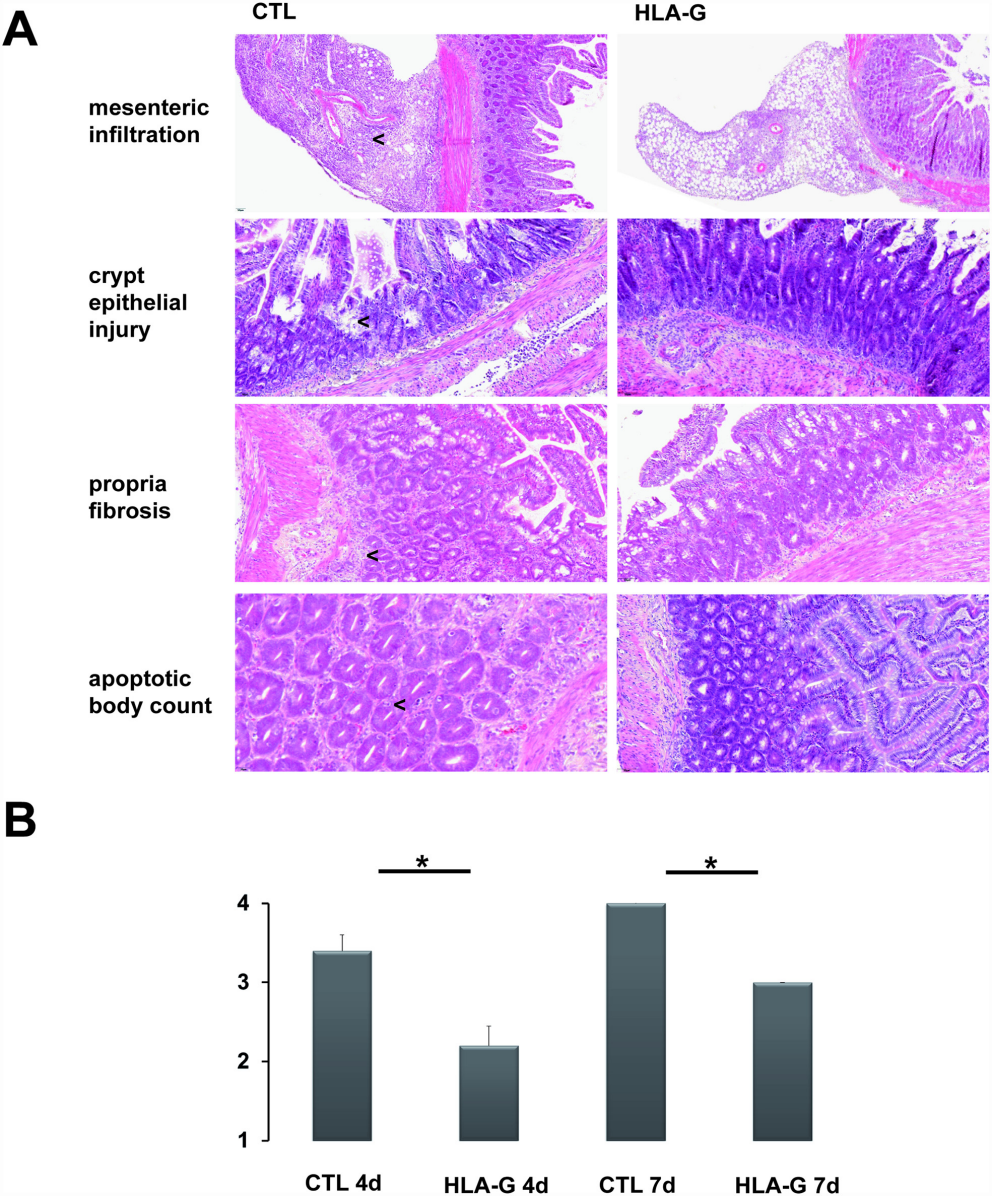
Morphologic characteristics and histologic grading of ACR using the Wu-score. (A) Representative H/E stained intestinal specimens with and without HLA-G treatment. Arrows indicate stronger leucocytic infiltration in graft mesentery, sites of crypt epithelial injury, propria fibrosis and increased crypt apoptotic body counts, respectively. (B) Overview of severity of ACR as assessed by Wu-score (one reviewer shown). n = 5 in each group.

Multiple formalin-fixed and paraffin-embedded graft sections of the mid-jejunum were obtained and stained with hematoxylin-eosin for histological grading. Specimens were blinded and graded for ACR by two independent reviewers (MW and ML) using the Wu score and standardized score sheets [19]. Interrater reliability of histopathologic assessment was confirmed using Cohen’s κ. At both time points (four and seven days), HLA-G treatment showed a significant reduction in ACR severity as assessed by the two independent reviewers. (CTL 4d vs. HLA-G 4d and CTL 7d vs. HLA-G 7d, Mann-Whitney U test: p<0.05, respectively). The interrater reliability showed a moderate agreement (Cohen’s κ = 0.57), see Fig 2A/2B. The increased rate of epithelial apoptosis associated with rejection, which was ameliorated by HLA-G treatment, was confirmed using TdT-mediated dUTP-biotin nick end labeling at POD7 (TUNEL, see S1 Fig).

### Myeloperoxidase-staining in graft muscularis whole-mounts by Hanker-Yates reaction

**Fig 3:**
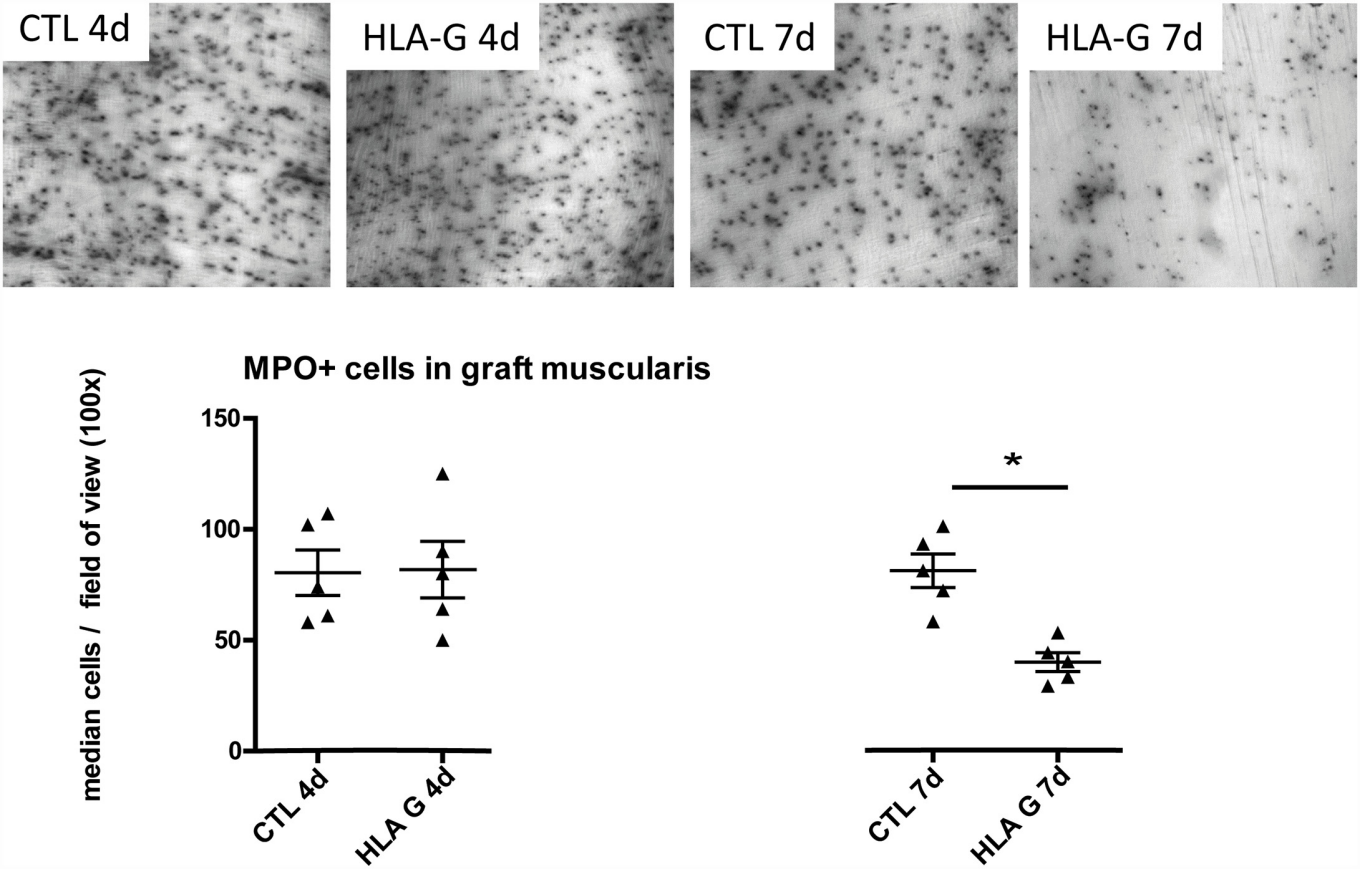
MPO^+^ cells in graft muscularis whole-mounts stained by Hanker-Yates reaction. At seven days after ITX, HLA-G treatment caused a significant reduction in MPO^+^ cell infiltrate in the graft muscularis.

To assess monocytic/granulocytic graft infiltration, graft muscularis whole mounts were freshly prepared from ileal segments by mechanical separation of the mucosa from the graft muscularis, fixing the tissue with 100% EtOH and staining the samples with Hanker-Yates reagent to visualize myeloperoxidase^+^-cell populations as described in [20]. Whole mounts were then analysed under a Nikon TE2000-E microscope, for the evaluation of myeloperoxidase^+^-cells, five fields of view (magnification 100x) were randomly chosen for cell count. The intestinal grafts showed a significantly reduced infiltration by MPO^+^ cells after HLA-G treatment at seven days after allogenic ITX compared to untreated controls (CTL 7d vs. HLA-G 7d, Mann-Whitney U test: p<0.05), see Fig 3. At the earlier timepoint of four days, MPO^+^ cell infiltration in the grafts did not differ between groups.

### In-vitro contractility measurements of isolated graft muscularis strips

**Fig 4:**
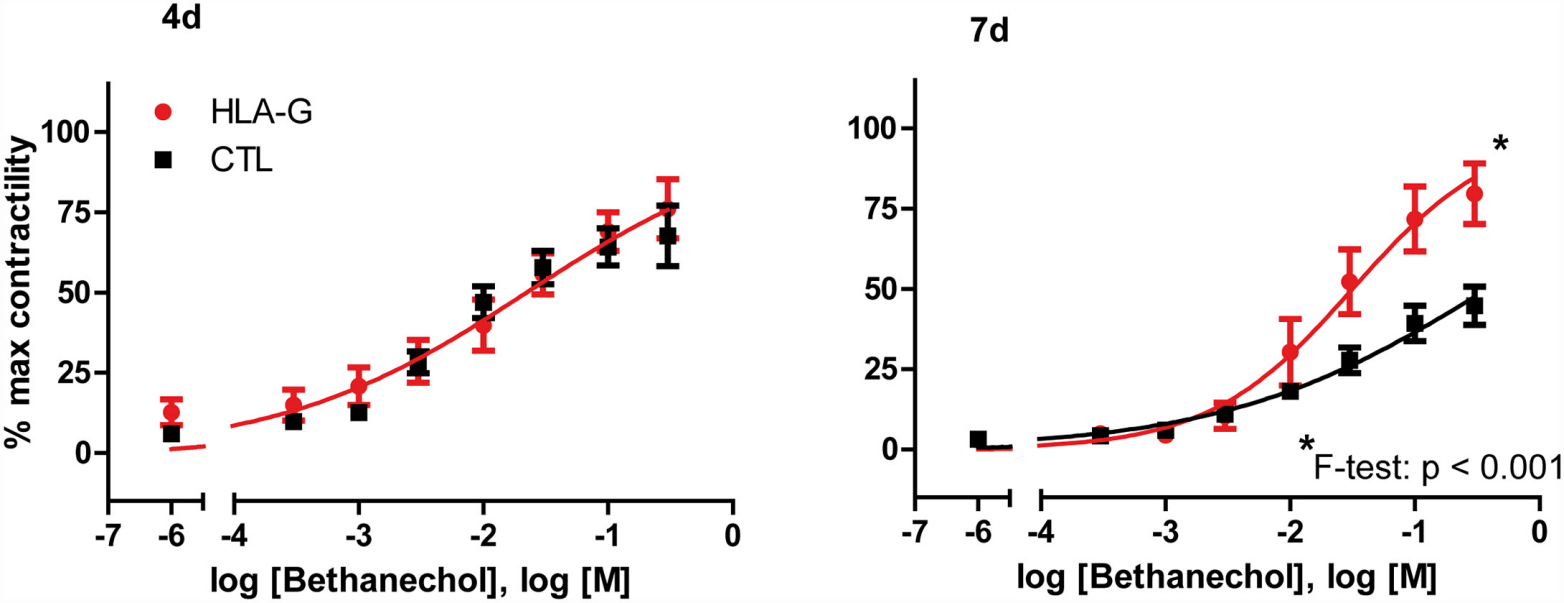
In-vitro graft contractility measured as a dose-response curve under betanechol stimulation. Dose response of graft contractility to rising bethanechol concentrations, curves fitted to a logistic four-parameter sigmoidal model. Both midpoint (logEC_50)_ and slope were significantly ameliorated by HLA-G treatment at seven days after ITX.

As a functional parameter for ACR-induced graft damage, in-vitro contractility of isolated muscularis strips in a standardized organ bath (oxygenated KRBS buffer) was analyzed by generating a dose-response curve using the muscarinic agonist betanechol in rising concentrations [3x10^-4^M—0,3M betanechol], as described in [20]. The inducible contractile force of separated graft muscularis strips is known to correlate with severity of ACR and structural integrity of the intestinal grafts [21]. In this case, betanechol-induced contractile force showed no difference at 4 days after transplantation with or without HLA-G treatment. However, at 7 days, a significantly ameliorated graft contractility with HLA-G treatment was observed (see Fig 4). After fitting the normalized data to a logistic curve as described above, the fitted midpoints (logEC_50_)/slopes were calculated and compared: logEC_50_ CTL 4d: -1.61 vs. logEC_50_ HLA-G 4d -1.69 (ns) and slope CTL 4d: 0.44 vs. HLA-G 4d: 0.44 (ns) or logEC_50_ CTL 7d: -0.41 vs. logEC_50_ HLA-G 7d -1.5 (significant, F test< 0.001) and slopes CTL 7d: 0.41 vs. HLA-G 7d: 0.76 (significant, F test< 0.001).

### Flow cytometric analysis

**Fig 5:**
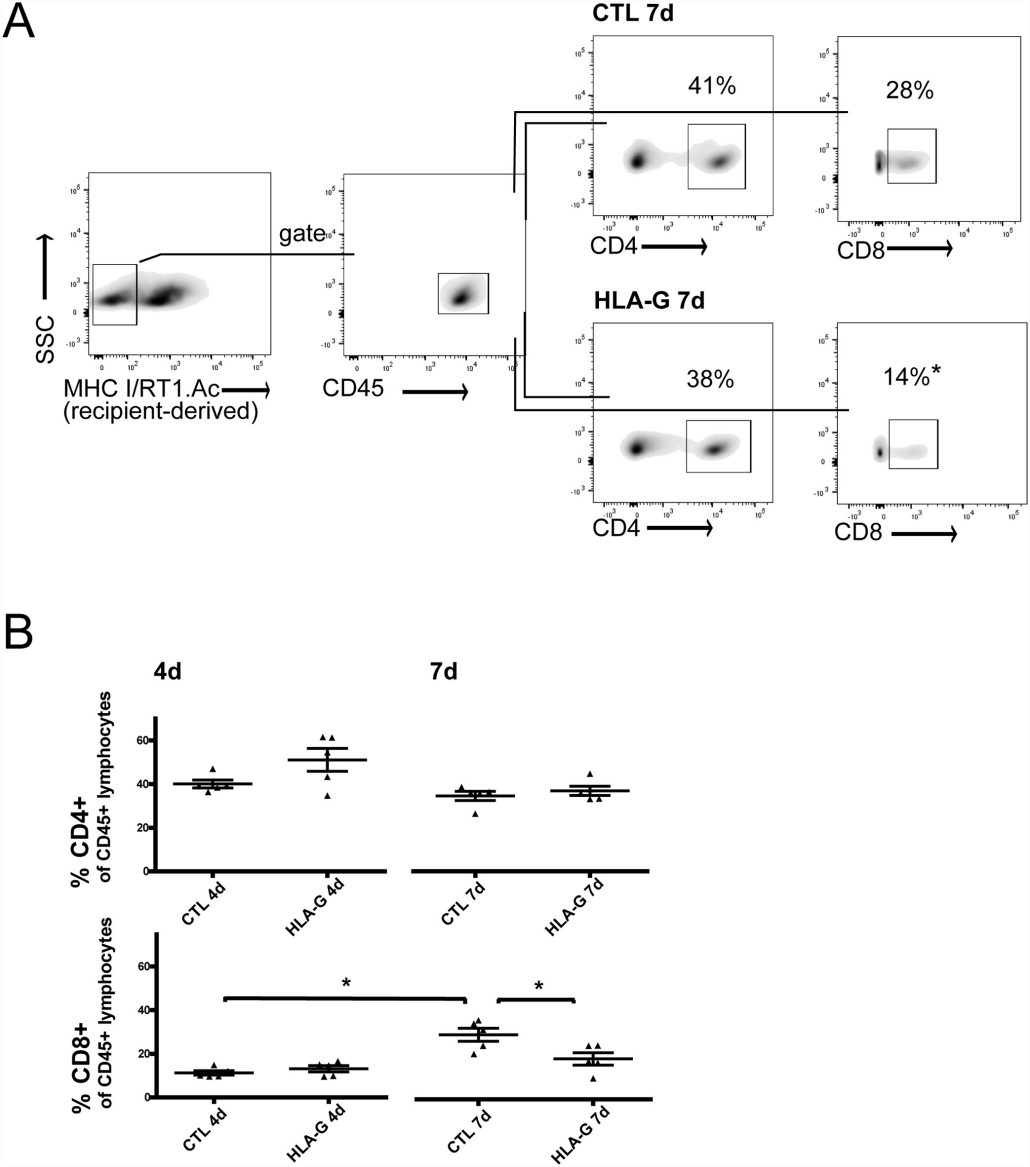
Gating strategy and overview of CD4^+^ and CD8^+^ T-cells isolated from gMLN with and without HLA-G treatment after allogenic intestinal transplantation. A) Representative gating strategy for recipient(LEW)-derived (MHC I/RT1.Ac-negative), CD45^+^ and CD8^+^ and likewise, CD4^+^ T-cells. Percentages of CD4^+^ and CD8^+^ are given as percentages of CD45^+^ parent population, shown is a representative result for CD8^+^ with and without HLA-G treatment at POD 7. (B) Overview of CD4^+^ and CD8^+^ T-cells with and without HLA-G treatment at both timepoints. A significant increase in CD8^+^ T-cells in the CTL groups is shown without treatment, as well as a significant reduction of this CD8^+^ T-cell population at day seven with HLA-G treatment.

**Fig 6:**
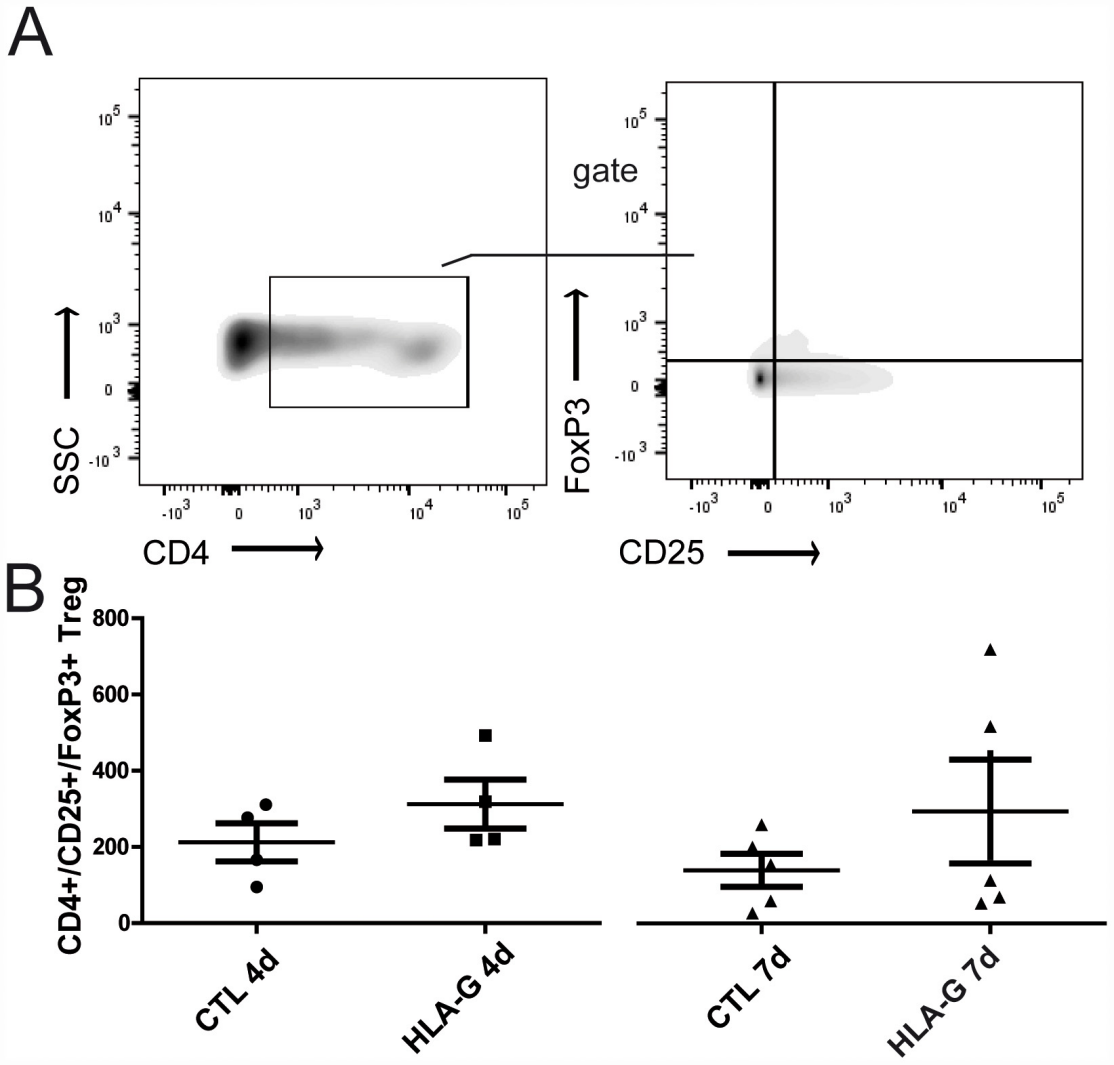
Gating strategy and overview of T_reg_ (CD4^+^/CD25^+^/FoxP3^+^) isolated from gMLN with and without HLA-G treatment after allogenic intestinal transplantation. (A) Gating strategy and representative result for Treg analysis. (B) Overview of classical Treg (CD4^+^/CD25^+^/FoxP3^+^ Treg), isolated from gMLN. A tendency to higher abundance in HLA-G treated animals, without reaching statistical significance (Mann-Whitney U test p>0.05), was observed.

After lymphocyte isolation fom the graft draining lymphnodes (gMLN), flow cytometric analysis was performed to assess the allogenic T-cell response and possible modulation by HLA-G treatment. Relative T-cell proportions of recipient-derived CD4^+^ and CD8^+^ are reported as percentages of the respective CD45^+^ parent population to allow individual comparison. At postoperative day four, no significant changes in either CD4^+^ or CD8^+^ cell infiltration of gMLN were observed with or without HLA-G treatment. At postoperative day seven however, ACR caused a significant rise in CD8^+^ cell infiltration in the CTL group versus the earlier timepoint of four days (CTL 4d vs. CTL 7d Mann-Whitney U test: p<0.05). Furthermore, this recipient-derived CD8^+^ cell infiltration in the gMLN was significantly reduced by HLA-G treatment compared to control (CTL 7d vs. HLA-G 7d, Mann-Whitney U test: p<0.05 (see Fig 5A/5B). This reduction of CD8^+^ cell infiltration was observed concomitantly to significantly ameliorated ACR in these grafts. Classical regulatory T-cells (CD4^+^/CD25^+^/FoxP3^+^ T_reg_), isolated from graft draining lymphnodes, showed a tendency to higher abundance in HLA-G treated animals, without reaching statistical significance (Fig 6, Mann-Whitney U test p>0.05).

### Real time quantitative PCR

**Fig 7:**
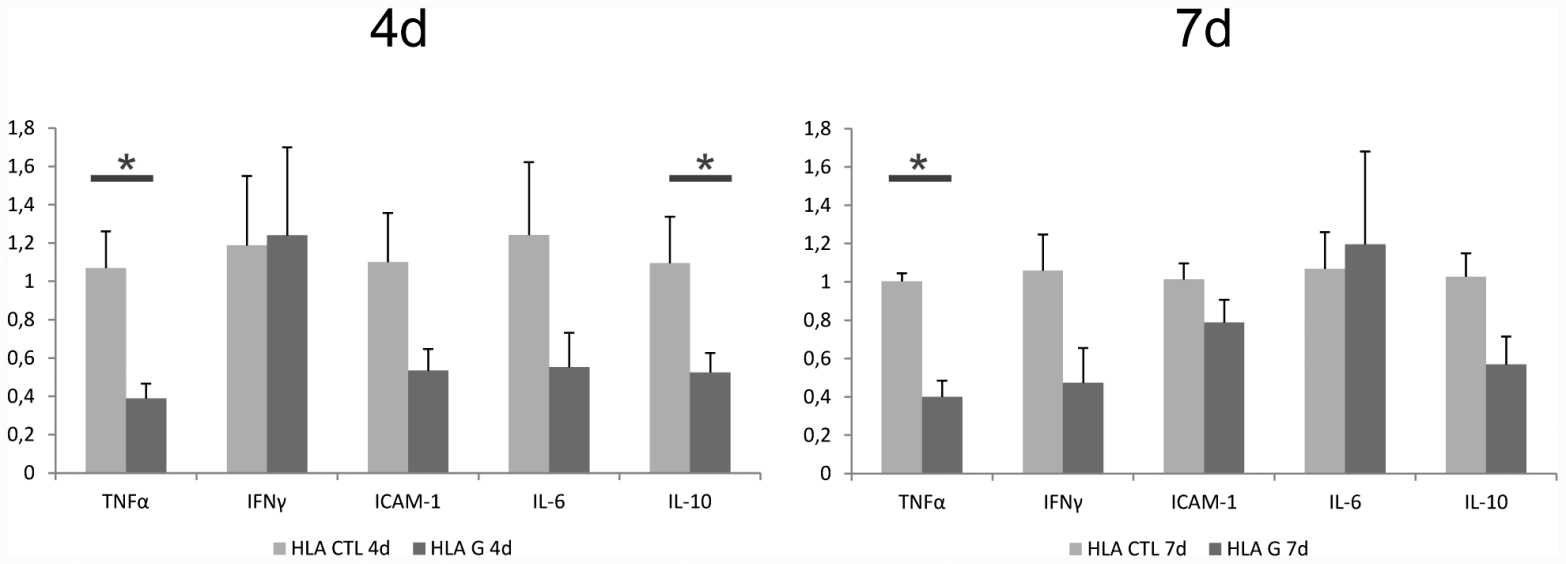
ACR-related gene expression in graft muscularis. The first time point at four days after allogenic ITX showed a significant downregulation of both TNFα and IL-10 by HLA-G treatment (Mann-Whitney U test p<0.05). At seven days after allogenic ITX, TNFα expression remained significantly reduced by HLA-G treatment.

To objectively assess the degree of molecular inflammation in the graft muscularis, qPCR based gene expression analysis of known ACR-related markers (TNFα, IFNγ, ICAM-1, IL-10 and IL-6) in graft muscularis samples was performed. Separated graft muscularis was obtained, snap-frozen immediatly in liquid nitrogen after organ harvest as described before, and mRNA was isolated and transcribed to cDNA using the commercially available kits mentioned above (Quiagen, Germany). The first time point four days after allogenic ITX showed a significant downregulation of both TNFα and IL-10 by HLA-G treatment (Mann-Whitney U test p<0.05). At seven days after allogenic ITX, TNFα expression remained significantly reduced by HLA-G treatment (see Fig 7). The presence of the Pir-B receptor (Lilrb3/NILR-1, Entrez Gene ID 65146) in the gMLN was confirmed using a specific FAM-labeled PCR probe as described above. The presence of the specific 77bp amplicon spanning an exon juction was confirmed in 4 different animals (triplicate detection) by gel staining (see S2 Fig), as previously described [36].

## Discussion

In this study, the immunosuppressive potential of recombinant HLA-G in the treatment of ACR after allogenic intestinal transplantation was demonstrated histologically and functionally. On the cellular level, a significantly reduced and ACR-related MPO^+^ -cell infiltration into the graft muscular layer and a reduced influx of recipient-derived CD8^+^ T-cells into the graft draining mesenteric lymphnodes were found as potential mechanisms of action. A relevant induction of classical CD4^+^/CD25^+^/FoxP3^+^ regulatory T-cells in the gMLN by HLA-G treatment could not be proven.

### Inflammation versus acute rejection

After intestinal transplantation, unspecific inflammation triggered by ischemia/reperfusion injury and the transplant process, occurs in the grafts within hours after reperfusion is started. These inflammatory processes impact on all compartments of the graft. The graft’s mucosal integrity is temporarily compromised as well as graft motoric function and structural changes in the graft tunica muscularis occur [22]. In this experiment, histologic grading of ACR using the Wu-score clearly showed that the HLA-G treated animals suffered from less severe ACR than untreated controls at both time points. The graft mucosal architecture was better preserved and crypt epithelial injury was less with HLA-G treatment as well as other morphologic aspects specified by the Wu-score (see Fig 2A). Morphologically, a distinct leucotic cell infiltration both in the lamina propria and the graft mesentery was identified in the H/E stained allograft specimens undergoing ACR as well a higher abundance of crypt apoptotic bodies, especially seven days after transplantation. The increased rate of epithelial apoptosis associated with rejection which was ameliorated by HLA-G treatment was confirmed using TdT-mediated dUTP-biotin nick end labeling on POD 7 (see S1 Fig). Overall, these morphologic features of ACR were markedly reduced in the groups treated with HLA-G.

Further analysis of graft whole mounts showed that a significant proportion of the observed leucocytic infiltrate consisted of MPO^+^ cells (see Fig 3). In the early postoperative phase, concomitant to the upregulation of inflammatory cytokines and chemotactic factors, an influx of MPO^+^-mono- and granulocytes along with an activation of the resident muscularis macrophages is a well described phenomenon in the graft muscularis [21]. Usually, these inflammatory changes resolve after a few days in the rat ITX model, if isogenic strain combinations (e.g. LEW->LEW) are used [23]. It is known that the mentioned MPO^+^ infiltrate correlates with ischemia/reperfusion injury and graft inflammation shortly after isogenic ITX and that this infiltrate intermittently resolves spontaneously. At day seven after allogenic ITX however, a persisting MPO^+^ infiltrate correlates strongly with the ongoing graft destruction caused by ACR [21]. In the allogenic model (BN->LEW) reported here, we likewise show the transplantation related influx of MPO^+^ leucocytes in the graft tunica muscularis especially at the early timepoint at day four and its persistence at the later timepoint at day 7 after allogenic ITX (see Fig 3). While day four is still within the timeframe of the regularly observed inflammatory changes in graft muscularis specimens and is not affected by HLA-G treatment, the MPO^+^ leucocytic infiltrate at POD seven must be largely attributed to ACR. Interestingly, HLA-G treatment caused a significant reduction of MPO^+^ cell infiltration in the graft muscularis at POD 7 (see Fig 3) possibly explaining the amelioration of ACR. Generally speaking, myeloid cells (e.g. neutrophils, but also monocytes and macrophages) stain MPO^+^ in the Hanker-Yates reaction and are either directly or indirectly affected by HLA-G treatment according to our data. Interestingly, the PIR-B inhibitory receptor which mediates HLA-G effects in the murine model, has previously been reported to be expressed on these myeloid cells which would support a direct interaction [24,25]. We indeed detected the Pir-B inhibitory receptor mRNA in the graft mesenteric lymphnodes in our model (see S2 Fig). While the protective effect of HLA-G at POD 7 can be explained by a reduced cell infiltration (of CD8^+^ cells and MPO^+^ cells) the early effects are less obvious. At POD 4, the cellular components of the infiltrate have not yet changed significantly, however the cytokine profile displays an early alteration with IL-10 and TNF-α downregulation. It could be assumed that the early tolerogenic effect produced by the HLA-G treatment could be effected by altering the cytokine profile of the cellular environment.

It is also known that ACR in intestinal allografts manifests itself not only histologically, but also functionally by exhibiting reduced in-vitro contractility ([21]). Our data shows that the observed histologic amelioration of ACR and reduced leucocytic cell infiltration by HLA-G treatment was reflected in functional analyses of graft contractility: A corresponding preservation of graft motor function was found at POD seven with HLA-G treatment (see Fig 4).

### ACR mediating T-cell populations

In the high-responder strain combination (BN->LEW) used for this study, a strong alloresponse results in severe ACR of the intestinal grafts without the use of immunosuppressive agents. It has been an ongoing debate which cell type is mainly responsible for this alloresponse in the setting of ITX. Both direct and indirect allorecognition play a role in intestinal transplantation as the transplanted intestine is abundant in “passenger” lymphocytes and antigen presenting cells (APCs). These donor-derived APCs can be directly recognized by- and in turn activate- recipient-derived T-cells. Likewise, donor antigens are shed in the circulation and reach the graft draining lymphnodes to be processed by recipient-derived APCs, which in turn can activate donor-specific T-cells [26]. To add complexity, a bidirectional lymphocyte trafficking to and from the grafted intestine, with recruitment of recipient monocytes, dendritic cells and other immunocompetent cells into the graft and likewise migration of donor-derived lymphoid cells into recipient tissues has been described [27]. It is therefore logical that the transplanted intestine is highly immunogenic and exclusive depletion neither of the CD8^+^, or CD4^+^ T-cell compartment or sole blockage of costimulatory pathways can completely prevent rejection. However, it is widely accepted that mainly influxing recipient-derived CD8^+^ T-cells and possibly NK cells cause ACR in this model [21,28,29]. Even though the rejection process may not be exclusively mediated by recipient-derived CD8^+^ T-cells [30], this population is widely accepted to play a major role in ACR [31]. From our data, we can now confirm that recipient-derived CD8^+^ T-cells are recruited into the gMLN in rising numbers as ACR progresses (CD8^+^ in group CTL 7d **>** CD8^+^ in group CTL 4), see Fig 5B. Furthermore, HLA-G itself is known to act a) via triggering CD8^+^ T-cell apoptosis by direct interaction with the CD8 T-cell receptor and activating the Fas/FasL pathway [9,10] and b) via tolerization of dendritic cells, leading to allogenic hyporesponsiveness and possibly to T_reg_-induction [14]. We therefore postulated that HLA-G should ultimately exhibit its tolerogenic characteristics by downregulation of the recipient-derived CD8^+^ T-cell population. We can now confirm this hypothesis in our model, as a significant downregulation of recipient-derived CD8^+^ T-cells was found with HLA-G treatment (CD8^+^ in group HLA-G 7d **<** CD8^+^ in group CTL 7d), see Fig 5A/5B. This reduction of recipient-derived CD 8^+^ T-cells was also clinically important because it correlated with ameliorated ACR, but whether this stemmed from induction of apoptosis in these T-cells or rather from a failure of T-cell activation/expansion due to HLA-G treatment (as has been described in vitro [32]), is still unclear at this point. Referring to a postulated T_reg_-induction, we could not prove an induction of the classical CD4^+^/CD25^+^/FoxP3^+^ T_reg_ population, (see Fig 6). Possible reasons for this failure to detect a significant T_reg_ induction is the scarcity of this T-reg subpopulation which may make it hard to detect in this in-vivo model. Another possibility would be that the phenotype of T_reg_ resulting from interaction with HLA-G (or from interaction with DCs exposed to HLA-G) may differ from the classical CD4^+^/CD25^+^/FoxP3^+^ T_reg_ phenotype, as HLA-G is known to also induce Tr1 cells and CD4low/CD8low T_reg_ independent of FoxP3 expression [33]. Thus, T_reg_ induction cannot be ruled out as a potential mechanism of action from our data and further research is warranted.

### Limitations of the study

A significant immunosuppressive effect of HLA-G treatment was seen in this model of experimental intestinal transplantation but it failed to completely resolve ACR. Arguably, a full tolerization was not expected in this high responder rat model without any classical immunosuppression given the high immunogenicity of the transplanted intestine. On the other hand, this high immunogenicity makes the intestine an interesting solid organ to study the effects of novel immunomodulatory drugs and biologicals as potential effects can be studied histologically and functionally in a sensitive system. Furthermore, although a significant effect on the recipient-derived CD8^+^ T-cell population was demonstrated, it is not deducible from our study whether this was due to induced T-cell apoptosis or failure of expansion/activation. Clearly further studies are needed to elucidate the mechanism of action of HLA-G in this model.

### Relevance of novel immunomodulatory strategies in intestinal transplantation

In the current era, only 60% of intestinal grafts remain functional after 4 years and chronic rejection is experienced by up to 20% of patients despite the ongoing advances in immunosuppressive therapy [34]. Historic approaches to achieve prope tolerance as well as modern immunosuppressive regimens based on monoclonal (anti-CD 25, anti-CD3, anti-CD52) or polyvalent (ATG, ALG) depleting antibodies combined with calcineurin inhibitors have not solved the problem of acute and especially chronic rejection. Until recently it was not feasible to test the tolerogenic potential of HLA-G paralogues in vivo. This has changed with the development of HLA-G fusion proteins such as the ß2-microglobulin fused to the HLA-G 5 heavy chain as described in [13] and [35]. Since the tolerogenic potential of HLA-G can now be explored for solid organ transplant research, this potentially creates new options for immunomodulation: Firstly, in combination with classical immunosuppression in order to achieve long term stable graft function and avoid chronic rejection and secondly, to minimize calcineurin-inhibitor-related toxicity and its long term adverse effects.

## Conclusion

This proof-of-principle study in an experimental model of orthotopic, allogenic intestinal transplantation demonstrates the immunoprotective effect of a recombinant HLA-G fusion protein for the first time in a solid organ transplant model. We show that HLA-G acts either directly or indirectly on an ACR-related and recipient-derived CD8^+^ T-cell population. Further studies on the in-vivo effects of HLA-G on T-cells, dendritic cells and T_reg_ subpopulations in the setting of acute and chronic rejection after solid organ transplantation are warranted.

## Supporting Information

S1 Fig TUNEL staining for detection of intestinal epithelial apoptosis. *An* increased rate of intestinal intraepithelial apoptosis was associated with rejection as detected using TdT-mediated dUTP-biotin nick end labeling with FITC immunofluorescence. In each animal, 5 random high power fields (20x) were chosen and intraepithelial apoptotic signals per 10 consecutive crypts were counted. HLA-G treatment showed significant reduction of intraepithelial apoptosis after 7 days (Mann-Whitney U test p<0,05).
(TIF)

S2 Fig Detection of the Pir-B receptor in the gMLN. In all 4 representative animals tested (triplicate tests), the presence of the specific Pirb receptor mRNA was detected in mRNA isolates from the gMLN using a specific probe for a 77bp amplicon as described above and in (36).
(TIF)
