# Supplementary figures and images for: Recombinant HLA-G as Tolerogenic Immunomodulant in Experimental Small Bowel Transplantation
Source: PLoS One. 2016 Jul 12;11(7):e0158907. doi: 10.1371/journal.pone.0158907 (PMC4942037; doi:10.1371/journal.pone.0158907)

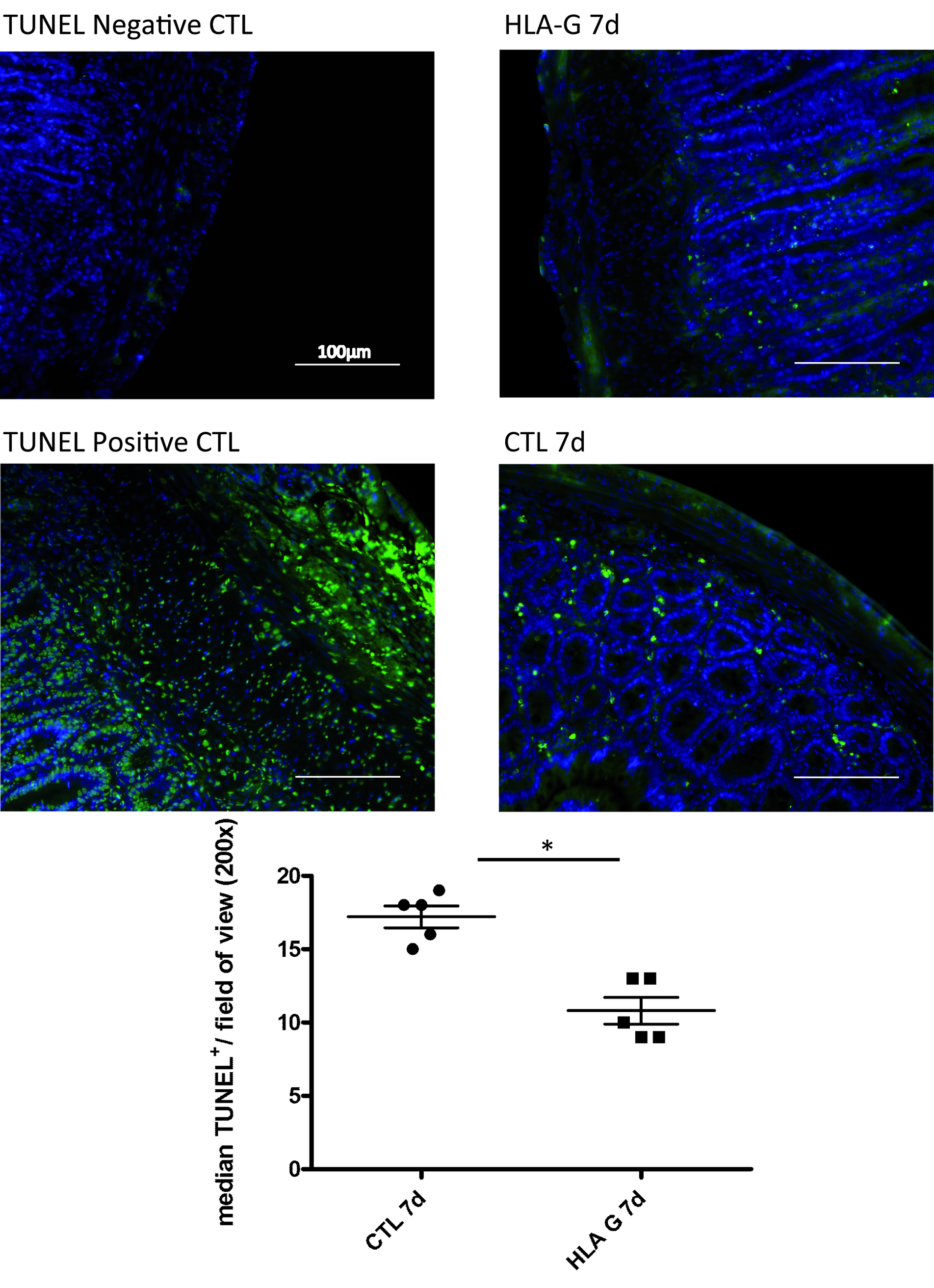

Supplement: S1 Fig — An increased rate of intestinal intraepithelial apoptosis was associated with rejection as detected using TdT-mediated dUTP-biotin nick end labeling with FITC immunofluorescence. In each animal, 5 random high power fields (20x) were chosen and intraepithelial apoptotic signals per 10 consecutive crypts were counted. HLA-G treatment showed significant reduction of intraepithelial apoptosis after 7 days (Mann-Whitney U test p<0,05). (TIF) [file pone.0158907.s001.tif]
